# Improving Initial Medication Adherence to cardiovascular disease and diabetes treatments in primary care: Pilot trial of a complex intervention

**DOI:** 10.3389/fpubh.2022.1038138

**Published:** 2022-12-06

**Authors:** Carmen Corral-Partearroyo, Alba Sánchez-Viñas, Montserrat Gil-Girbau, María Teresa Peñarrubia-María, Ignacio Aznar-Lou, Antoni Serrano-Blanco, Cristina Carbonell-Duacastella, Carmen Gallardo-González, Maria del Carmen Olmos-Palenzuela, Maria Rubio-Valera

**Affiliations:** ^1^Health Technology Assessment in Primary Care and Mental Health (PRISMA) Research Group, Institut de Recerca Sant Joan de Déu, Esplugues de Llobregat, Spain; ^2^Department of Paediatrics, Obstetrics, Gynaecology and Preventive Medicine, Univ Autonoma de Barcelona, Bellaterra, Spain; ^3^Consortium for Biomedical Research in Epidemiology and Public Health (CIBER en Epidemiología y Salud Pública), Madrid, Spain; ^4^Facultat de Medicina i Ciències de la Salut, Universitat de Barcelona, Barcelona, Spain; ^5^Parc Sanitari Sant Joan de Déu, Sant Boi de Llobregat, Spain; ^6^Research Network on Chronicity, Primary Care and Health Promotion (RICAPPS), Barcelona, Spain; ^7^Primary Care Centre Bartomeu Fabrés Anglada, Direcció D'Atenció Primària Regió Metropolitana Sud, Institut Català de la Salut, Barcelona, Spain; ^8^Unitat de Suport a la Recerca Regió Metropolitana Sud, Fundació Institut Universitari per a la Recerca a l'Atenció Primària de Salut Jordi Gol i Gurina (IDIAPJGol), Barcelona, Spain; ^9^Facultat de Farmàcia, Universitat de Barcelona, Barcelona, Spain

**Keywords:** primary care, complex intervention, shared decision-making (SDM), medication adherence, pilot, feasibility study

## Abstract

**Introduction:**

The Initial Medication Adherence (IMA) intervention is a multidisciplinary and shared decision-making intervention to improve initial medication adherence addressed to patients in need of new treatments for cardiovascular diseases and diabetes in primary care (PC). This pilot study aims to evaluate the feasibility and acceptability of the IMA intervention and the feasibility of a cluster-RCT to assess the effectiveness and cost-effectiveness of the intervention.

**Methods:**

A 3-month pilot trial with an embedded process evaluation was conducted in five PC centers in Catalonia (Spain). Electronic health data were descriptively analyzed to test the availability and quality of records of the trial outcomes (initiation, implementation, clinical parameters and use of services). Recruitment and retention rates of professionals were analyzed. Twenty-nine semi-structured interviews with professionals (general practitioners, nurses, and community pharmacists) and patients were conducted to assess the feasibility and acceptability of the intervention. Three discussion groups with a total of fifteen patients were performed to review and redesign the intervention decision aids. Qualitative data were thematically analyzed.

**Results:**

A total of 901 new treatments were prescribed to 604 patients. The proportion of missing data in the electronic health records was up to 30% for use of services and around 70% for clinical parameters 5 months before and after a new prescription. Primary and secondary outcomes were within plausible ranges and outliers were barely detected. The IMA intervention and its implementation strategy were considered feasible and acceptable by pilot-study participants. Low recruitment and retention rates, understanding of shared decision-making by professionals, and format and content of decision aids were the main barriers to the feasibility of the IMA intervention.

**Discussion:**

Involving patients in the decision-making process is crucial to achieving better clinical outcomes. The IMA intervention is feasible and showed good acceptability among professionals and patients. However, we identified barriers and facilitators to implementing the intervention and adapting it to a context affected by the COVID-19 pandemic that should be considered before launching a cluster-RCT. This pilot study identified opportunities for refining the intervention and improving the design of the definitive cluster-RCT to evaluate its effectiveness and cost-effectiveness.

**Clinical trial registration:**

ClinicalTrials.gov, identifier NCT05094986.

## Introduction

The prevalence of non-initiated pharmacological treatments ranges from 2 to 40%, varying between medications and contexts and depending on patient characteristics and motivations (1–3). Non-initiation of chronic treatments, such as those for cardiovascular disease (CVD) and diabetes, generates a high burden on the healthcare system, which is aggravated by poor adherence (2, 4–8). Reducing non-initiation and improving long-term adherence is, therefore, a priority (9). Previous studies have evaluated interventions to reduce non-initiation but none of these interventions were theory-based and most of the studies showed a high risk of bias (10–15). To date, few interventions have focused on shared decision-making (SDM) strategies to improve adherence, which present promising results regarding improved health outcomes (16–19).

Carefully designing and piloting an intervention improves the likelihood of its effectiveness, transferability and sustainability (20, 21), especially in the case of complex interventions such as those aiming to change patients' and healthcare professionals' behavior. The Non-Initiation project followed the Medical Research Council (MRC) framework for complex interventions to gain an in-depth understanding of this behavior and contribute to the appropriate use of medications in primary care (PC) (20). Between 2014 and 2019, phase I, or the development phase, was carried out and epidemiological studies and qualitative research with patients and healthcare professionals were conducted to understand initiation behavior and design the Initial Medication Adherence (IMA) intervention (22–27). It is a complex, multidisciplinary, SDM intervention to improve initiation, secondary adherence, and clinical parameters in patients who receive a new prescription for CVDs or diabetes in PC. As per the non-initiation model (25, 26), the intervention works on two levels: the patient's intrapersonal level, based on the empowerment of the patient by increasing health literacy and SDM (28–30); and the patient's interpersonal level, based on the interaction between the patient and healthcare professionals, and their support (31–33). The intervention includes decision aids that target patients >18 years old with a risk of CVD and diabetes and were designed in collaboration with healthcare professionals.

This paper describes the results of phase II, or feasibility phase, which aimed to evaluate the feasibility and acceptability of the IMA intervention, the feasibility of the evaluation study, a pragmatic cluster-Randomized Controlled Trial (34, 35), and to ultimately optimize the IMA intervention and its evaluation design. The specific aims were to (1) test the availability and quality of data used to assess the effectiveness and cost-effectiveness of the IMA intervention, (2) evaluate the feasibility and acceptability of the IMA intervention in PC, and (3) revise and redesign the intervention decision aids.

## Materials and methods

### Study design

This pilot study was a cluster non-randomized controlled trial with an embedded process evaluation. The availability and quality (completion rate and reliability) of Real-World Data (RWD) records of the pilot trial were explored (aim 1), recruitment and retention rates were estimated and intervention group participants were interviewed (aim 2) and discussion groups with PC patients were conducted to review and redesign the decision aids (aim 3).

The results of this study are reported according to the Consolidated Standards of Reporting Trials (CONSORT) extension to pilot and feasibility trials (36).

**Figure 1:**
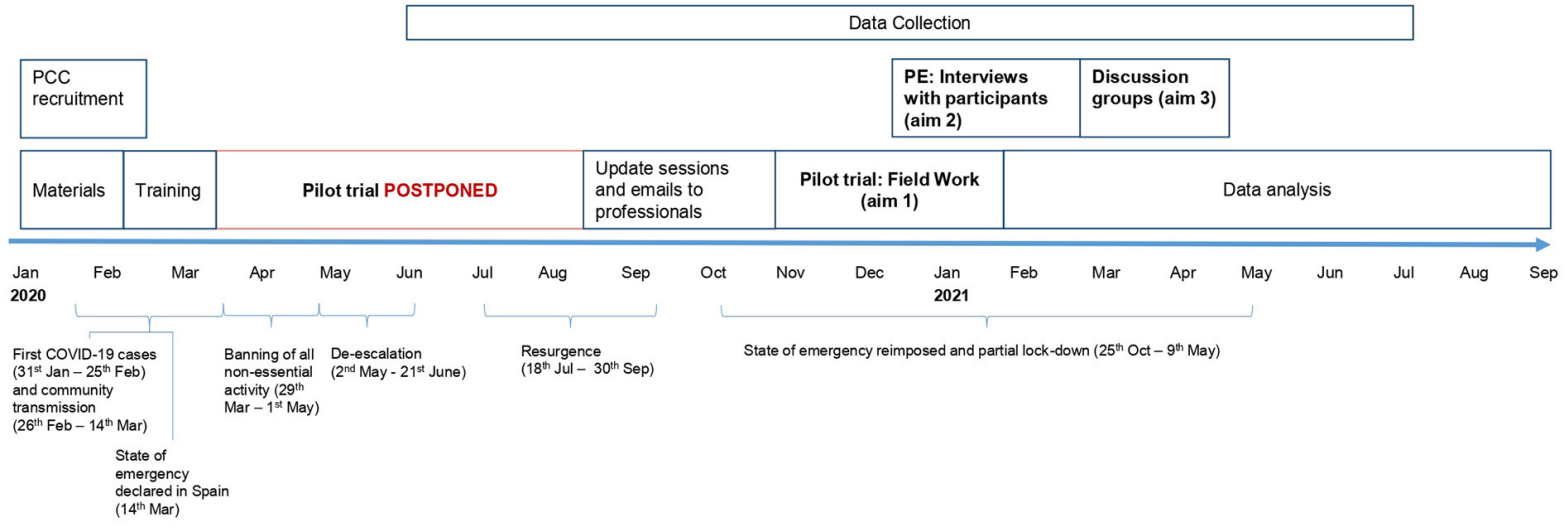
Timeline of the pilot study and COVID-19 periods in Spain. PCC, primary care center; PE, process evaluation. Aim 1: To test the availability and quality of data used to assess the effectiveness and cost-effectiveness of the IMA intervention. Aim 2: To evaluate the feasibility and acceptability of the IMA intervention in PC. Aim 3: To review the intervention decision aids to ultimately redesign them.

Figure 1 shows the timeline of the pilot study, which was affected by the Coronavirus Disease (COVID-19) pandemic and the need to adapt it to this context. The intention was to carry out the intervention from March 2020 to May 2020, but adaptations were applied and it was finally launched in November 2020 and continued until January 2021.

### Setting

Healthcare in Spain is based on universal coverage for all citizens with free access at the point of use (with some exceptions) and is mostly funded by taxes (37). PC is the gatekeeper of the healthcare system, providing healthcare, health education, prevention activities, and community services. It consists essentially of a team of general practitioners (GP), nurses, and social workers, who are based in PC centers. Patients have an assigned GP and nurse. Prescription medicines are dispensed in community pharmacies by pharmacists who have access to the electronic prescription system (37). Patients can fill a prescription at any community pharmacy. The e-prescription system includes a warning that alerts the pharmacist to first prescriptions of inhalers, platelet aggregation inhibitors, anticoagulation, and insulin treatments.

### Pilot study

#### Participants and group assignment

A convenience sample of five PC centers in Catalonia (Spain) participated in the study. GPs and nurses at the selected PC centers, together with pharmacists from community pharmacies in the reference area of the PC centers, were invited to participate. Professionals that agreed to participate provided signed informed consent. No other inclusion criteria were applied.

The study targeted patients (>18 years old) who received a new prescription of antihypertensive, lipid-lowering, antiplatelet, and/or antidiabetic (oral and/or insulin) medications. A prescription was considered new in the absence of prescriptions for medications of the same pharmacotherapeutic group during the previous 6 months. Patients' informed consent was obtained by simplified means (see “Ethics statement”) (38). No other inclusion criteria were applied.

Using convenience criteria, two PC centers were assigned to the control group and three to the intervention group. Healthcare professionals and patients were classified into intervention and control groups according to the reference PC centers and due to the nature of the intervention; professionals and patients were not blind to it.

Sample size calculation was not estimated prior to the pilot trial, although the sample was designed to be representative of the target cRCT population and was based on the same inclusion/exclusion criteria (39).

#### Description of intervention

The IMA intervention standardizes care and provides knowledge, skills, and tools to GPs to promote SDM when prescribing a new treatment for CVDs or diabetes, and to nurses and pharmacists to explore patients' doubts and offer supplementary information, promoting consistency and coordination of care. By applying the principles of SDM the patient is encouraged to express their concerns and preferences and actively participate in the decision process at their preferred level (29, 30). The implementation strategy has two main inputs: *training for professionals* on the motives underlying non-initiation, communication skills, health literacy, SDM, and the use of the decision aids; and *decision aids* (leaflets and a website) with information on the disease and treatment options to increase patients' health literacy and support SDM. The GP delivers the intervention at least once during the prescription process. Nurses and pharmacists deliver intervention on patients' demand during follow-up consultations and medication dispensing.

No training or decision aids were provided to professionals in the control group, who were asked to provide care as usual.

The IMA intervention was designed to be applied during face-to-face consultations, yet it was adapted to the COVID-19 context during the pilot study. When the new treatment was prescribed by phone, the GP emailed the leaflet contents to patients, and/or they were invited to collect it at the pharmacy. Additionally, the GP or nurse phoned the patient a week after the prescription to check whether questions had arisen.

#### Availability and quality of RWD for the trial (aim 1)

##### Trial outcomes and data collection

The primary trial outcome was initiation, defined as having a dispensing record following a new prescription (the index prescription) (40). A single prescription filled was considered an alternative outcome for initiation in sensitivity analysis. Secondary outcomes included implementation, clinical parameters [systolic and diastolic blood pressure, total cholesterol, low-density lipoprotein, high-density lipoprotein, blood glucose, glycated hemoglobin, estimated glomerular filtration rate, and cardiovascular risk (41)] and costs (use of healthcare services and days of sick leave).

Other variables included patient characteristics (sex, age, and diagnosis) and PC center characteristics according to non-initiation predictors (22): reference population, type of center (resident-training center or not), and socioeconomic status of the area divided into four urban categories based on quartiles, from low (urban 4) to high (urban 1), and a rural category.

All data were obtained from electronic health records registered at the public primary healthcare system database in Catalonia (Institut Català de Salut; ICS): System for the development of Research in Primary Care (SIDIAP) (42). Data were extracted for the follow-up period from June 2020 to June 2021.

##### Analysis

Descriptive analysis (counts, proportions, and means) was conducted using Stata 17 to explore all available variables and identify missing data and outliers.

First, the sociodemographic profile of the PC centers and participants at a prescription level (a patient can have more than one new prescription) was described.

Secondly, initiation was assessed by considering the time of prescription at the PC center and the dispensing month at the community pharmacy. Non-initiation was defined as not having collected the treatment prescribed (i.e., absence of dispensing records) within 3 months after the index prescription. A single prescription filled was defined as one dispensation only during the follow-up period. Costs were measured by taking into account the use of healthcare services, which included visits to PC professionals (GP or nurse), secondary care referrals, diagnostic tests, and days of sick leave. We assessed the reliability of recorded visits to PC professionals by calculating the proportion of new prescriptions and clinical parameters with a visit record on the same day.

Thirdly, the quality of clinical parameter records in the electronic health records was assessed. We calculated clinical parameter values and the proportion of prescribed treatments that had a clinical parameter registered during the follow-up period following care quality standards based on clinical practice guidelines (43–46).

#### Feasibility and acceptability of the IMA intervention (aim 2)

A process evaluation was integrated into the pilot study, collecting quantitative and qualitative data to measure professional recruitment and retention rates, assess the context and implementation of the IMA intervention in terms of fidelity to study protocol and the COVID-19 pandemic, and describe professionals' and patients' experiences and perceptions of the intervention in terms of feasibility and acceptability.

##### Quantitative data collection and analysis

Professional recruitment rates were registered in study forms before the pilot trial (March 2020) and after the trial was stopped and restarted (November 2020). Those professionals recruited in November were interviewed to estimate retention rates.

We used descriptive statistics (frequency and proportion) to estimate professionals' recruitment and retention rates.

##### Qualitative data collection and analysis

Following purposive sampling criteria, all the professionals and a selection of patients from the intervention group were invited to participate in the process evaluation. The research team contacted nineteen GPs, three nurses, and sixteen pharmacists by phone and email. GPs from the intervention group contacted five patients and invited them to participate in the study and to be interviewed by a researcher. All the participants signed informed consent prior to the interview.

Semi-structured telephone interviews with professionals were performed during and after the study was completed using a topic guide based on the intervention and the health theories and models it is based on (range 15–25 min). Field notes were made during and after the call. To increase the validity of the results, answers were summarized at the end of the interview and participants were asked to validate them.

Semi-structured face-to-face and telephone interviews with patients followed a topic guide based on the intervention and their intention to initiate the new medication after the intervention (range 20–40 min). These were recorded, anonymized, and transcribed by the research team.

Field notes and transcripts from semi-structured interviews were included as narrative data and analyzed following the principles of thematic content analysis (47) by two qualitative researchers. Data were organized and grouped by professionals and patients. Firstly, the researchers familiarized themselves with the data by re-reading notes and listening to recordings. Each researcher created a coding framework following a deductive and inductive approach. Open coding was applied to the data and codes were then organized into themes as per the research questions, based on pre-existing categories of the intervention, and new categories extracted about the mechanisms of action and context of the intervention and the attitude of patients regarding their pathology and treatment. Coding frameworks were triangulated, and themes were reviewed and refined by the two researchers before applying them to all the data.

#### Redesign of the IMA intervention tools (aim 3)

Patients from the PC system in Catalonia were recruited following a maximum variation sampling strategy based on some of the predictors of non-initiation: nationality, age, educational level, and presence of CVD and diabetes risk (22). Twenty-four patients were contacted. Patients that agreed to participate provided signed informed consent.

##### Data collection and analysis

Three discussion groups (duration 90–120 min) were conducted with four to six participants using a topic guide based on the protocol and IMA intervention decision aids, focusing particularly on health literacy and SDM. Discussion groups were recorded, anonymized, and transcribed by the research team.

Discussion groups were analyzed following a thematic analysis approach (47) by four researchers. Firstly, the researchers familiarized themselves with the data by listening to the recordings. Comments of the discussion groups were transcribed and rearranged to follow the intervention protocol, pre-existing categories of the decision aids, and new categories involving these tools that arose in the discussion groups. For each category, the main ideas were coded and reviewed to determine themes and identify patterns and, finally, the findings were triangulated between the researchers. No new themes emerged after coding the second discussion group.

## Results

### Participants

**Figure 2:**
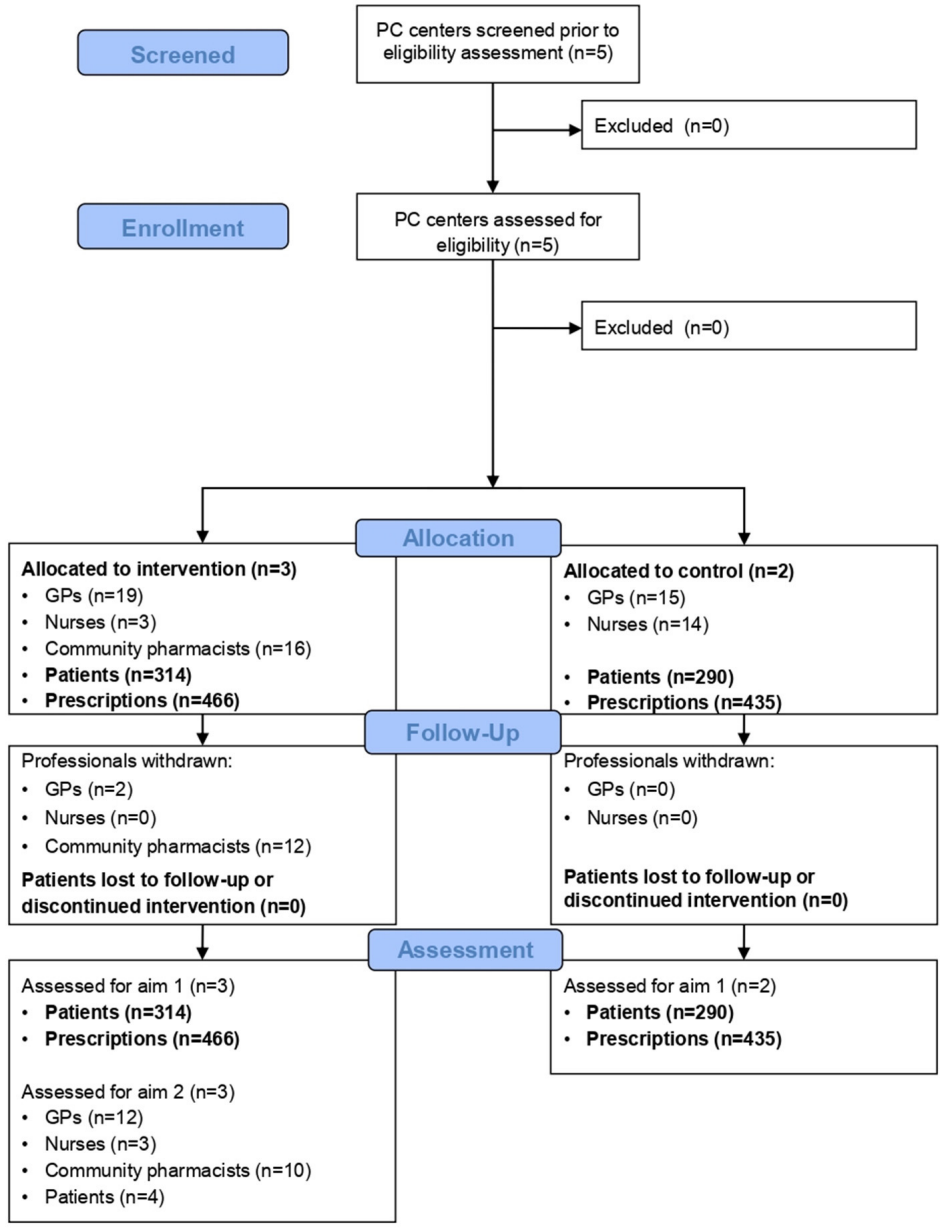
CONSORT Flow diagram (36). GP, general practitioner; PC, primary care. Aim 1: To test the availability and quality of data used to assess the effectiveness and cost-effectiveness of the IMA intervention. Aim 2: To evaluate the feasibility and acceptability of the IMA intervention in PC.

During the pilot trial, 901 new treatments of antihypertensive, lipid-lowering, antiplatelet, and/or antidiabetic (oral and/or insulin) medications were prescribed to 604 patients, 314 in the intervention group (see Figure 2 for details on recruitment and follow-up).

Tables 1, 2 show the characteristics of the participant PC centers and patients. PC centers were located in urban areas with different socioeconomic status, size and proportion of immigrant population and most were training centers (Table 1). Half of the medications were prescribed to women (50.83%), with the mean age of patients being 62.6 years old. Most of the prescriptions had a diagnosis record (89.7%); with the highest frequency being hypertensive disease (59.6%). There were almost no differences between women and men in terms of age and diagnoses, except for diabetes (Table 2).

**Table 1:** Characteristics of the PC centers.

	**Training center**	**Area socioeconomic status^a^**	**Reference population^b^ (*N*)**	**Immigrant population (%)**
**Intervention group**
PCC1	No	Urban 4	10,174	7.15%
PCC2	Yes	Urban 4	20,299	37.33%
PCC3	Yes	Urban 4	26,782	33.41%
**Control group**
PCC4	Yes	Urban 3	26,094	11.07%
PCC5	Yes	Urban 2	14,092	13.41%

PCC, primary care center.

^a^Socioeconomic status: four urban categories based on quartiles from low (urban 4) to high (urban 1) socioeconomic level and a rural category.

^b^Number of people assigned to the Primary Care Center (48).

**Table 2:** Characteristics of the patients*.

**Prescriptions**	**Women (*N* = 458)**	**Men (*N* = 443)**	**Total (*N* = 901)**
**Age (mean, SD)**	64.15 (16.22)	61.01 (15.21)	62.60 (15.80)
**Diagnosis records (ICD-10)**	404 (88.21%)	406 (91.24%)	808 (89.68%)
Diabetes mellitus type 2 (E10–E14)	151 (32.97%)	212 (47.86%)	363 (40.29%)
Dyslipidemia (E70–E90)	220 (48.03%)	197 (44.47%)	417 (46.28%)
Hypertensive diseases (I10–I15)	275 (60.04%)	262 (59.14%)	537 (59.60%)
Ischemic heart diseases (I20–I25)	30 (6.55%)	58 (13.09%)	88 (9.77%)
Other heart diseases (I30–I52)	29 (6.33%)	33 (7.45%)	62 (6.88%)
Cerebrovascular diseases (I60–I69)	68 (14.85%)	46 (10.38%)	114 (12.65%)
Arterial diseases (I79–I79)	15 (3.28%)	29 (6.55%)	44 (4.88%)
Glomerular diseases (N00–N08)	4 (0.87%)	3 (0.68%)	7 (0.78%)
Acute and chronic kidney failure (N17–N19)	52 (11.35%)	58 (13.09%)	110 (12.21%)
**No diagnosis records^a^**	54 (11.79%)	39 (8.80%)	93 (10.32%)

SD, standard deviation; ICD, International classification of diseases (49).

*Patients characteristics are described using prescription level as a unit of analysis.

^a^Absence of intervention-related diagnosis records in the electronic health records.

The process evaluation involved 12 GPs, three nurses, 10 pharmacists, and four patients. Two GPs declined the invitation to participate due to time restrictions, and the rest failed to reply. One patient declined to participate in the study. Over half of the professionals were women, ranging between 41 and 52 years old and with more than 10 years of experience in PC. Half of the patients were women, ranging between 50 and 68 years old, and they were prescribed different medications and had different work and educational levels. Finally, 15 patients from the PC system in Catalonia agreed to participate in the discussion groups, varying by sex, age, cardiovascular risk, and educational level. Characteristics of the participants are shown in Supplementary Tables 1–3.

### Availability and quality of RWD for the trial (aim 1)

#### Initiation and implementation

These variables have no missing data. In total, 10.7% of prescriptions were not initiated 3 months after the index prescription, and 18.4% were single prescriptions filled.

Table 3 summarizes indicators of data availability and quality for clinical parameters. Missing records in patient electronic health records were >50% in all cases before the index prescription, and between 39.7% (systolic and diastolic blood pressure) and 85.2% (cardiovascular risk) after the index prescription, with the lowest being cardiovascular risk in both cases. All parameter values were within plausible ranges except one estimated glomerular filtration rate CKD-EPI value which was recorded manually.

**Table 3:** Data availability and quality for clinical parameters for baseline (pre-prescription) and follow-up (post-prescription) assessment.

**Medication prescribed and clinical parameter**	**Prescriptions**	**Missing records pre-prescription**	**Missing records post-prescription**	**Records**	**Clinical parameter values**	**Clinical parameter values**
	***N***	***N* (%)**	***N* (%)**	***N***	**Mean (SD)**	**Range**
**Antihypertensive^a^**	406	277 (68.23%)	161 (39.66%)			
Systolic blood pressure (mmHg)		277 (68.23%)	161 (39.66%)	797	138.17 (20.25)	85; 230
Diastolic blood pressure (mmHg)		277 (68.23%)	161 (39.66%)	798	81.55 (12.65)	45;129
**Lipid-lowering^b^**	199	118 (59.30%)	147 (73.87%)			
High-density lipoprotein (mg/dl)		118 (59.30%)	147 (73.87%)	433	55.89 (15.17)	21; 106
Low-density lipoprotein (mg/dl)		118 (59.30%)	147 (73.87%)	432	114.61 (40.86)	31; 244
Total cholesterol (mg/dl)		59 (29.65%)	123 (61.81%)	681	200.21 (52.30)	70; 489
**Antidiabetic^c^**	191	95 (49.74%)	108 (56.54%)			
Blood glucose (mg/dl)		71 (36.79%)	78 (40.41%)	847	119.84 (48.21)	62; 486
Glycated hemoglobin (%)		84 (43.52%)	89 (46.11%)	368	7.10 (1.61)	4.3; 15.3
Estimated glomerular filtration rate		84 (43.52%)	100 (51.81%)	1,629		
MDRD (mL/min/1.73 m^2^)				161*	46.79 (11.78)	12.9; 59.9
CKD-EPI (mL/min/1.73 m^2^)				512*	68.38 (18.06)	0.4; 89.9
**All prescriptions^d^**	901	842 (93.45%)	768 (85.24%)			
Cardiovascular Risk (REGICOR %)						

^a^Pharmacotherapeutic groups: C02 Antihypertensives, C03 Diuretics, C07 Beta blocking agents, C08 Calcium channel blockers, and C09 Agents acting on the renin-angiotensin system.

^b^Pharmacotherapeutic groups: C10 Lipid modifying agents.

^c^Pharmacotherapeutic groups: A10 Drugs used in diabetes.

^d^Pharmacotherapeutic groups: A10 Drugs used in diabetes, B01 Antithrombotic agents, C02 Antihypertensives, C03 Diuretics, C07 Beta blocking agents, C08 Calcium channel blockers, C09 Agents acting on the renin-angiotensin system, and C10 Lipid modifying agents.

*Estimated Glomerular Filtration Rate MDRD and CKD-EPI appear as >60.1 and >90.1, respectively, in the electronic health records for normal values. We have considered only those values below 60.1 and 90.1 to assess the quality of the records. For more details, please refer to Supplementary material.

Tables 4, 5 summarize indicators of data availability and quality for use of services and productivity losses. A 33.3% of prescriptions didn't have a visit registered on the day of a new prescription, while there were 13.8–27% of clinical parameter measures without any visit records on the same day (Table 4). After the index prescription all values for healthcare services and productivity losses were within plausible ranges, and no outliers were detected (Table 5).

**Table 4:** Data availability of visits the day prescription*s* were issued and clinical parameters were measured.

**Medication prescribed**	**Prescriptions**	**Missing visits on the day of prescription**
	***N***	***N* (%)**
Antihypertensive^a^	406	125 (30.79%)
Lipid-lowering^b^	199	65 (32.66%)
Antidiabetics^c^	191	60 (31.41%)
All prescriptions^d^	901	300 (33.30%)
**Clinical parameter**	**Records**	**Missing visits on the day of measure**
	***N***	***N*** **(%)**
Systolic blood pressure	797	113 (14.18%)
Diastolic blood pressure	798	114 (14.29%)
High-density lipoprotein	433	67 (15.47%)
Low-density lipoprotein	432	66 (15.28%)
Total cholesterol	681	119 (17.47%)
Blood glucose	847	229 (27.04%)
Glycated hemoglobin	368	54 (14.67%)
Estimated glomerular filtration rate	1,629	439 (26.95%)
Cardiovascular risk (REGICOR %)	159	22 (13.84%)

^a^Pharmacotherapeutic groups: C02 Antihypertensives, C03 Diuretics, C07 Beta blocking agents, C08 Calcium channel blockers, and C09 Agents acting on the renin-angiotensin system.

^b^Pharmacotherapeutic groups: C10 Lipid modifying agents.

^c^Pharmacotherapeutic groups: A10 Drugs used in diabetes.

^d^Pharmacotherapeutic groups: A10 Drugs used in diabetes, B01 Antithrombotic agents, C02 Antihypertensives, C03 Diuretics, C07 Beta blocking agents, C08 Calcium channel blockers, C09 Agents acting on the renin-angiotensin system, and C10 Lipid modifying agents.

**Table 5:** Data quality for use of services (number of services used) and productivity losses (number of days of sick leave) (*N* prescriptions = 901).

	**Mean (SD)**	**Range**
**Use of services**
GP visits	6.45 (4.77)	0; 34
Nurse visits	4.54 (5.97)	0; 59
Secondary care referrals	0.14 (0.41)	0; 3
Diagnostic tests	0.21 (0.52)	0; 4
**Productivity losses**
Days of sick leave	5.61 (24.83)	0; 152

GP, general practitioner; SD, standard deviation.

### Feasibility and acceptability of the IMA intervention (aim 2)

#### Professional recruitment and retention rates

Table 6 shows the professional recruitment and retention rates. Overall, recruitment was lower for nurses than for GPs and pharmacists. Retention was the highest for GPs and nurses. Only two GPs were lost due to sick leave. Low retention rates of pharmacists were attributed to the study being postponed and the COVID-19 distance measures in place.

**Table 6:** Professional recruitment and retention rates.

	**Professionals**	**Recruitment**	**Retention^a^**	
	***N***	***N* (%)**	***N* (%)**	
		February 2020	November 2020	
**Intervention group**
PCC1	GPs (8)	5 (62.50%)	5 (62.50%)	5 (100%)
	Nurses (7)	7 (100%)	3 (42.86%)	3 (100%)
	Community pharmacies (8)	8 (100%)	8 (100%)	3 (37.50%)
PCC2	GPs (15)	10 (66.67%)	10 (66.67%)	9 (90%)
	Nurses (12)	3 (25%)	0 (0%)	N/A
	Community pharmacies (5)	4 (80%)	4 (80%)	1 (25%)
PCC3	GPs (15)	4 (26.67%)	4 (26.67%)	3 (75%)
	Nurses (12)	0 (0%)	0 (0%)	N/A
	Community pharmacies (6)	4 (66.67%)	4 (66.67%)	0 (0%)
**Control group**
PCC4	GPs (14)	7 (50%)	7 (50%)	7 (100%)
	Nurses (13)	8 (61.54%)	7 (53.85%)	7 (100%)
PCC5	GPs (10)	8 (80%)	8 (80%)	8 (100%)
	Nurses (9)	7 (77.78%)	7 (77.78%)	7 (100%)

GP, general practitioner; N/A, not applicable; PCC, primary care center.

^a^Retention rate based on the professionals recruited in November 2020.

#### Context and implementation of the IMA intervention

The COVID-19 pandemic influenced the implementation of the IMA intervention and fidelity to the study protocol. Training was completed long before the pilot was finally carried out, and professionals described more consultations for acute health problems, fewer follow-up and preventive consultations and therefore fewer chronic medication prescriptions. All along with an increased workload at both PC centers and pharmacies. All professionals described an increase in telephone consultations and, as a result, an increase in the duration of face-to-face consultations (reporting ~15 min per patient). Nevertheless, different practices within different organizations were reported. One of the PC centers in the intervention group had returned to face-to-face consultations by November 2020, whereas the other two were doing mainly telephone consultations. In the case of community pharmacies, most had increased the physical distance from patients due to the pandemic.

**Figure 3:**
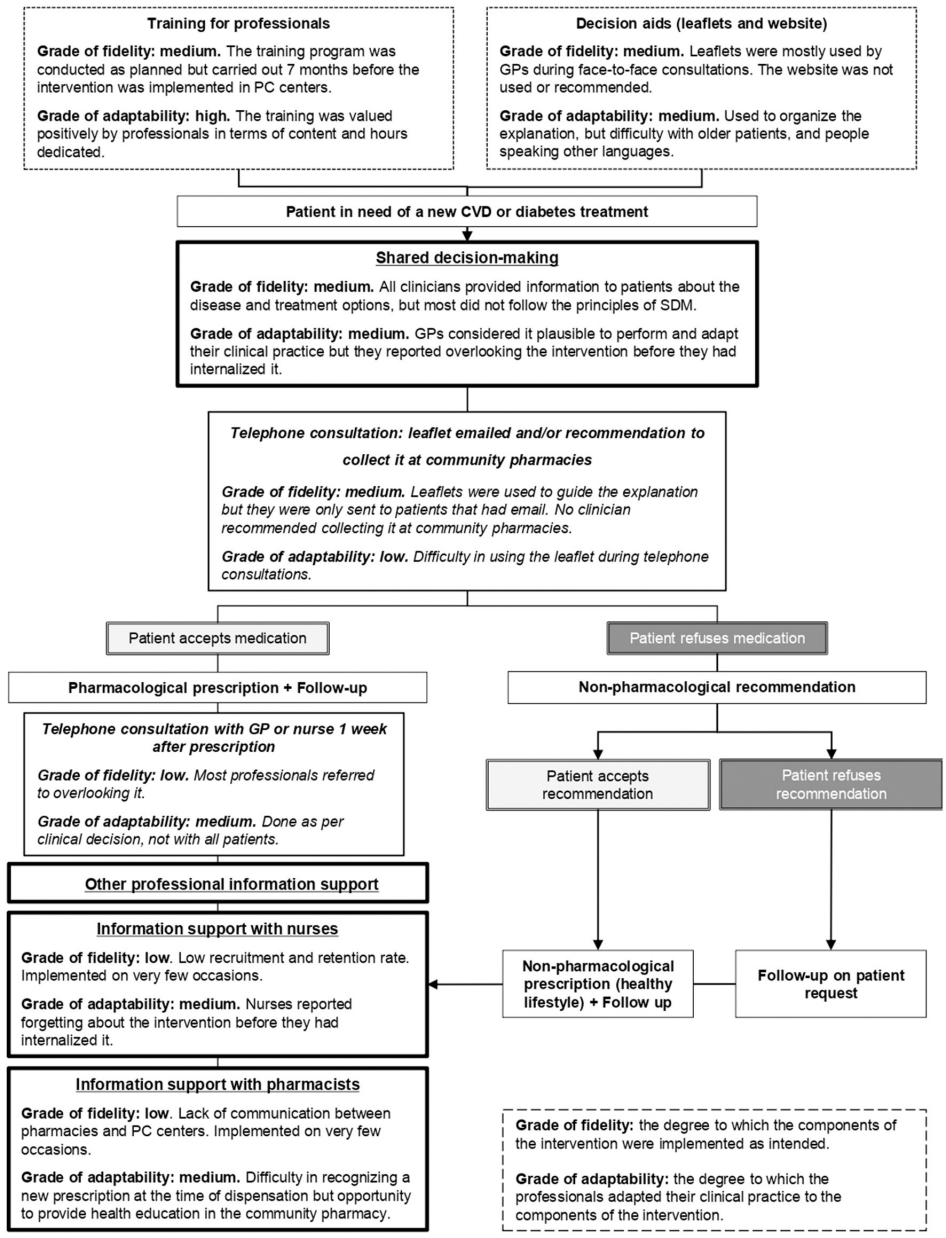
Grade of fidelity and adaptability to the implementation strategy and processes of the IMA intervention. CVD, cardiovascular disease; GP, general practitioner; PC, primary care. Text in italics describes the intervention adaptations made due to the COVID-19 pandemic.

The implementation strategy and processes of the IMA intervention, contextual factors, and the grade of fidelity to the study protocol and grade of adaptability to the intervention are described below and summarized in Figure 3.

##### Training for professionals

The training was generally valued positively in terms of content and hours dedicated. Professionals understood non-initiation as a public health problem, GPs recognized situations in which the patient accepted a new prescription during a consultation but never initiated it, and appreciated the tools provided during training to approach new prescriptions. Nevertheless, due to the delay of the pilot study, some GPs and nurses and most pharmacists, mentioned that they had forgotten about it.

##### Decision aids

PC professionals agreed that the leaflet was helpful in organizing the information given to patients. However, some found it challenging and questioned its utility when used with older patients and people who did not speak Spanish or Catalan. Most of the pharmacists reported not using the leaflets, and none of the professionals reported using the website or recommending it to patients.

GPs considered it was easier to implement the intervention face-to-face using the leaflets than by telephone consultations. Those that implemented it by telephone used the leaflet to guide themselves through the explanation and sent it online only to those patients that had email. Three out of four patients stated that GPs used a leaflet during the explanation of the new prescription, one of them through telephone consultation. In the last case, the leaflet was sent by email and the GP phoned the patient some days later to ensure the information was understood.

##### Shared decision-making

At the time of a new prescription, GPs considered that the intervention was easy to apply and adapted their clinical practice accordingly. They mainly reported applying the intervention during face-to-face consultations and having enough time to do so. Providing information to the patient about the disease and treatment options was considered part of the standard practice of the GP, and all of them reported doing so. Nonetheless, only two GPs reported following the principles of SDM when recommending a new medication. The majority stated that the patient agreed with the prescription, and only two mentioned that the patient decided with them to issue the prescription.

Of the patients that stated that the GP provided information using the leaflet, only one reported SDM during the prescribing process. In the other cases, the GP did not ask their opinion or preferences and prescribed the medication only after they explained the disease and the treatment. When patients were asked about participating in the decision process, some of them considered it was not a decision for them to make. Some considered they need not be involved because of a lack of knowledge in the field but also because they trusted the GP's decision.

##### Other professional information support

Both GPs and nurses considered the fact that few nurses participated in the study to be a barrier to the intervention. Nurses were believed to have an important role in the follow-up and identification of patients with CVDs or diabetes. Additionally, professionals at the PC centers and pharmacists cited a lack of communication between one another. Pharmacists were often not considered as part of the multidisciplinary PC team, which was seen as a barrier to implementing the intervention at all levels; GPs as prescribers, and nurses and pharmacists as central supporters.

Most nurses and pharmacists participating reported implementing the intervention on very few occasions, and none of the patients interviewed confirmed that the nurse or the pharmacist implemented the intervention with them. Some visited the nurse after the prescription for a follow-up on the chronic disease and all mentioned that the pharmacist dispensed the medication without any explanation.

Broadly, the main barrier to implementing the intervention was forgetfulness. Professionals tended to overlook it before they had internalized it as their standard practice. In addition, pharmacists found it difficult to recognize a new prescription at the time of dispensation, especially if the alert on the e-prescription system was not available.

#### Professionals' and patients' experiences and perceptions in terms of feasibility and acceptability: Key themes

Summarized below are the key themes regarding feasibility and acceptability, such as the experiences and perceptions of the GPs as prescribers, nurses, and pharmacists as key supporters, and patients as recipients of the IMA intervention.

##### Perceived effect of the IMA intervention by professionals

Professionals believed that, even though the information was very similar to that of usual care, patients understood it better when the leaflet was used to structure the information and considered this could have a direct impact on adherence. A negative effect in terms of initiation was related to giving more information about medication adverse effects to patients with chronic conditions with no symptoms. Some professionals believed patients may be more afraid of adverse effects than future complications associated with the disease.

##### Relationship and trust between the professional and patient

Trust in professional recommendations was perceived to be affected by the relationship between the professional and the patient, which was considered to be mainly influenced by the length of time the patient had visited the same professional. Trust was described as the main facilitator. From the professional's point of view, it makes it easier to maintain a conversation with the patient and explore their perceptions, while from the patient's perspective, it makes it easier to ask questions and express their opinion.

##### Motivation for professionals to adapt their clinical practice

Even though most professionals described the COVID-19 pandemic as a difficult situation, some GPs emphasized they were more willing to make changes as they considered the IMA intervention as reinforcement of the importance of SDM in their routine practice. Similarly, pharmacists saw it as an opportunity to provide health education in the community pharmacy, especially to those patients that were not able to visit the PC center during the pandemic.

### Redesign of the IMA intervention tools (aim 3)

PC patients highlighted the advantages and disadvantages of the pilot leaflets according to their needs. As for disadvantages, they emphasized a lack of topic titles to introduce the content, the medical jargon, and the large amount of information provided. As advantages, they highlighted the structure of the leaflet and specific contents such as the epidemiological data on the disease, data on the consequences of the decision not to treat, and the encouragement to express their doubts and opinions and participate in the decision process.

Moreover, patients recommended that the new leaflets should clarify whether the non-pharmacological measures are an alternative to the medication or an addition to it, so the patient is encouraged to adopt non-pharmacological measures in the case of a pharmacological prescription. Additionally, patients suggested that only the most common adverse effects of the medication should be mentioned so that the risk-benefit assessment of the medication is balanced.

Patients acknowledged they looked on the internet when they had questions about their disease or treatment after consultation with clinicians. However, they found it very difficult to find a website that was reliable and supported by official organizations, and with easy-to-understand content. With respect to the website that was being designed for the definitive trial, they considered it should have links to other patients' associations, as well as to the Catalan Electronic Health System, so they had the option to contact a PC professional directly if they had any queries.

## Discussion

The results of this pilot study suggest that implementing an intervention based on SDM to improve adherence to medications for CVDs and diabetes in PC is feasible and that the intervention is well-accepted. Carrying out a pragmatic cRCT to evaluate the effectiveness and cost-effectiveness of such an intervention is also feasible but weaknesses in the study design and the implementation of the intervention were identified and the knowledge gained should be used to refine the intervention and the study (50).

A non-initiation rate of 11% is in line with previous studies that were used to calculate the sample size of the cRCT (22, 27). The study identified weaknesses in the electronic health records by recognizing a high prevalence of missing registered visits. This could be explained, in the context of the COVID-19 pandemic, and by an increased number of telephone and emergency consultations (51). At the time of the pilot study the workload in PC centers was high, which could partly explain flaws in data records. This is not expected to happen during the cRCT, but if missing visit records are identified, and taking into account that all prescriptions would be issued in PC centers in the public health system, every prescription would be imputed as one visit to the GP so costs are not underestimated. Additionally, there was a high proportion of missing clinical parameter records that could be explained by the COVID-19 situation, when face-to-face visits were kept to the minimum, and by the short follow-up period (5 months). Care quality standards based on clinical practice guidelines from the Catalan Health System recommend taking measurements at least every 12 months for all parameters except cholesterol, which is recommended every 18 months (43–46). During the training stage for professionals, the importance of registering clinical parameters according to clinical practice guidelines will be reinforced to reduce the percentage of missing data obtained through RWD during the trial. However, values of the parameters were mainly within the expected range. Special attention will be paid to records entered manually that are expected to increase during the cRCT. The sample size of the trial exceeded the estimates determined in previous feasibility study research (39, 52, 53).

The COVID-19 pandemic impacted the recruitment of nurses and the retention of pharmacists, as professionals reported, although recruitment rates of PC professionals were already low in some centers in February 2020, especially in the case of nurses. Other studies have also identified difficulties in recruitment and retention rates of healthcare professionals in PC, particularly due to lack of time, high workload, and low engagement with the research topic (54, 55). To improve professional recruitment rates and promote participation, before contacting PC professionals, we will inform stakeholders of PC and pharmacy organizations in Catalonia, as well as managers and directors of PC centers. Furthermore, the IMA-cRCT will be presented in a short session to professionals at each selected PC center and pharmacy, and they will be given time to ask the research team questions and deliberate participation in the study. Additionally, the research team will contact professionals participating in the trial regularly to troubleshoot, provide support, and therefore improve retention.

In general, professionals failed to apply the principles of SDM and both professionals and patients perceived some of the barriers and facilitators that have previously been cited in the literature (56, 57). For instance, professionals reported overlooking the intervention and both professionals and patients questioned patients' willingness to get involved in the decision process. However, patient preferences for SDM are influenced by the perception of professionals regarding SDM and its approach when inviting the patient to take part in the process (29, 56). Professionals recognized that SDM could increase patients' knowledge and improve adherence to medications, and even though time has been reported as a barrier before (56), none considered time to be a restriction to applying the intervention in this study. SDM is the foundation of the IMA intervention, involving patients in the decision process empowers them and increases self-efficacy by increasing health literacy and awareness of their pathologies and treatment options, and therefore the potential to increase adherence to treatment plans (17, 30). Patients are invited to express their opinions and if they decide not to start the medication the prescription is not issued. Likewise, they are actively involved in the treatment follow-up, information on medication effects and adverse events is given so patients can take them into account in the decision-making process as well as identify them and act accordingly if the treatment is initiated. To increase professionals' understanding and engagement with SDM, the training will be extended to 6 h, with 3 h dedicated to SDM. To balance professionals' schedules, it will be divided into two sessions. Session one would cover non-initiation as a public health problem and the development of the IMA intervention, as well as its practical aspects, such as records and ethical requirements. Session two will focus on communication skills and SDM and this preparation has been designed by an expert in the field. All professionals will be trained together to increase cohesion between GPs, nurses, and pharmacists, and reinforce the role of the latter two in providing information and supporting the patient in the decision process when a new chronic pharmacological treatment is prescribed.

The main advantages and disadvantages of the decision aids were identified and will be used to redesign and respect the preferred information format for patients as recommended by SDM models (29). The leaflets will contain essential information written in plain language, with a clear distinction between non-pharmacological measures and pharmacological treatments, and a section encouraging patients to express their opinion and professionals to write recommendations to patients. Additionally, they will be translated into the most widely-spoken languages in Catalonia. The content of the website will be appraised by healthcare organizations in Catalonia and the layout will be designed to make it more user-friendly. It will be divided into pathologies and pharmacological treatments and the leaflets will be easier to acquire as patients and professionals will be able to download them from the website.

The COVID-19 pandemic has inevitably impacted the implementation of the intervention during the pilot study. However, not all the consequences were negative. As described by professionals, the pandemic encouraged them to adapt their clinical practice to new situations and reinforced the role of pharmacists in providing health education. Additionally, the duration of face-to-face consultations was increased, which might have favored the implementation of the IMA intervention. Organizational changes during the COVID-19 pandemic and the reintroduction of usual practices in PC centers and pharmacies would need to be considered carefully during the implementation of the IMA intervention in a pragmatic PC setting during the upcoming cRCT.

Some limitations need to be acknowledged. First, the duration proposed for this pilot study was 3 months of fieldwork and 6 months of follow-up before and after the index prescription. However, due to the COVID-19 pandemic, the duration of the follow-up period dropped to 5 months, which might have impacted the access to parameter data in the electronic health records. Second, the study was only carried out in one region of Catalonia in the context of a pilot study, and even though PC centers had dissimilar socioeconomic characteristics, the results obtained might have been different if various regions of Catalonia had been included. Third, the low recruitment rate of nurses, especially after the COVID-19 pandemic, might have limited the assessment of the role of nurses in the IMA intervention. Lastly, not all the professionals who participated in the trial were interviewed and we might have missed some important insights. Nevertheless, the percentage of participation among professionals was high, all were invited to participate and had the opportunity to be interviewed at their preferred date and time.

Involving patients in the decision-making process is fundamental in achieving better clinical outcomes, although patient-centered care requires modifications to clinical practice in PC. We identified barriers and facilitators to implementing the intervention as well as adapting it to a context affected by the COVID-19 pandemic. This pilot study contributes information regarding the feasibility and acceptability of the IMA intervention and its evaluation design in a pragmatic setting. It has helped to identify strengths and weaknesses and refine the IMA intervention and its evaluation design accordingly before the definitive cRCT to evaluate the effectiveness and cost-effectiveness of the IMA intervention.

## Data availability statement

The datasets presented in this article are not readily available because the research team is not the quantitative data owner as it only analyzes information that is property of public health institutions. The data that support the findings of this study are available from SIDIAP but restrictions apply to the availability of these data, which were used under license for the current study, and thus are not publicly available. However, data are available from the authors upon reasonable request and with the permission of SIDIAP. Qualitative data and the study protocol are available from the authors upon reasonable request. Requests to access the datasets should be directed to maria.rubio@sjd.es.

## Ethics statement

This study was reviewed and approved by the Drug Research Committee (CEIm) at the IDIAP Jordi Gol, codeCEIm 19/198-P. The pilot study is a low-intensity intervention clinical trial where groups of subjects are allocated to the control and the intervention group. Informed consent was obtained by simplified means which requires that the same information stated under Article 30 of Regulation (EU) No 536/2014 is provided before anyone is enrolled in the trial, and after being informed, the patient does not object to participating (38). All conditions described in Regulation (EU) No 536/2014 and the Real Decreto 1090/2015 were fulfilled (38, 58). Informed consent in the present study was obtained by displaying posters in prominent locations of the participating PC centers notifying people that a clinical trial was being conducted in the center and that patients could be part of this comparative study. Posters contained information on how and why the trial was being conducted and what the implications of participating in the study were. Furthermore, professionals in the intervention and control groups were trained to deal with patients' queries regarding the study. If patients declined to participate in the study, this information was documented by clinicians in the electronic health records and data from those patients was not used for the study. Finally, patients could withdraw at any time from the study without any detriment.

Participation in this study was entirely voluntary. Professionals that agreed to participate signed informed consent at the training session and agreed to be interviewed as part of the process evaluation. Patients who participated in the process evaluation and discussion groups signed informed consent after recruitment and prior to the beginning of data collection. All participants had the right to refuse to participate and to withdraw from the study at any time.

## Author contributions

MR-V led the design of the study and obtained funding for the study. IA-L, MG-G, and MP-M advised and contributed to the study design. MR-V, IA-L, MG-G, MP-M, and CC-D designed the decision aids. MR-V, IA-L, MG-G, and MP-M recruited and trained professionals. CC-P, AS-V, IA-L, and MR-V developed the statistical analysis plan and analyzed quantitative data. CC-P, MG-G, MP-M, and AS-V collected and analyzed qualitative data. CC-P wrote the first draft of the manuscript. All authors added to and approved the final manuscript.

## Funding

The project IMA-cRCT has received funding from the European Research Council (ERC) under the European Union's Horizon 2020 research and innovation programme (GA No. 948973) from January 1st 2021. Thanks to this grant we can apply the results to adapt the final intervention and its assessment and disseminate the results of the study that was funded by a research grant from the College of Pharmacists of Barcelona (Col·legi de Farmacèutics de Barcelona) in 2019 in order to develop the intervention, the fieldwork and the analysis. IA-L had a CIBERESP contract (CIBER in Epidemiology and Public Health, CB16/02/00322) during the development of this study. MP-M has the 14th ICS support for the promotion of group research strategies through the intensification of researchers (7Z22/009) and 16th ICS support for the promotion of group research strategies through the intensification of researchers (7Z20/028), from the IDIAP Jordi Gol. CG-G has the 17th ICS support for the promotion of group research strategies through the intensification of researchers (7Z21/019), from the IDIAP Jordi Gol. CC-D had a PFIS research contract both from the Institute of Health Carlos III (ISCIII), Ministry of Economy and Competitiveness (Spain) (FI20/00007) when the study was developed.

## Conflict of interest

The authors declare that the research was conducted in the absence of any commercial or financial relationships that could be construed as a potential conflict of interest.

The handling editor declared a shared research network (Research Network in Chronicity, Primary Care and Health Promotion RICAPPS) with the authors MG-G, MO-P, and CG-G at the time of the review.

## Publisher's note

All claims expressed in this article are solely those of the authors and do not necessarily represent those of their affiliated organizations, or those of the publisher, the editors and the reviewers. Any product that may be evaluated in this article, or claim that may be made by its manufacturer, is not guaranteed or endorsed by the publisher.

## Abbreviations

CONSORT: Consolidated Standards of Reporting Trials
COVID-19: Coronavirus Disease
cRCT: cluster-Randomized Controlled Trial
CVD: Cardiovascular Disease
GP: General Practitioner
MRC: Medical Research Council
IMA: Initial Medication Adherence
PC: Primary Care
RWD: Real-World Data
SDM: Shared Decision Making
SIDIAP: System for the development of Research in Primary Care.
