# Supplementary material for: Improving Initial Medication Adherence to cardiovascular disease and diabetes treatments in primary care: Pilot trial of a complex intervention
Source: Front Public Health. 2022 Dec 6;10:1038138. doi: 10.3389/fpubh.2022.1038138 (PMC9764337; doi:10.3389/fpubh.2022.1038138)
Supplement: Supplementary file 2 [file Data_Sheet_1.docx]

Supplementary Material

Supplementary Table 1. Process evaluation: Professionals' characteristics

| Primary Care Centers (PCC) | Professional category | Sex | Age range (mean) | Years of experience in PC | Supplementary information |
| --- | --- | --- | --- | --- | --- |
| PCC1 | GP: 3  Nurse: 3  Pharmacists: 5 (employees)* | Women: 10  Men: 1 | 25-50 (41.4) | < 10: 2  10-20: 4  >20: 5 | *No training center* |
| PCC2 | GP: 6  Pharmacists: 1 (owner)* | Women: 4  Men: 3 | 42-60 (51.7) | < 10: 1  10-20: 3  >20: 3 | *Training center*  Resident supervisors: 3 |
| PCC3 | GP: 3  Pharmacists: 4 (2 owners and 2 employees)* | Women: 7 | 34-59 (48.7) | < 10: 1  10-20: 2  >20: 4 | *Training center*  Resident supervisors: 2 |

PC: Primary care; PCC: Primary Care Center; GP: General Practitioner

*In Spain, the owner of a pharmacy can only be a pharmacist. A pharmacy can have up to two owners, which may be pharmacists and many pharmacy employees including pharmacists and pharmacy technicians.

Supplementary Table 2. Process evaluation: Patients' characteristics

| Patients | Sex | Age | Nationality | Medication prescribed | >1 CVD or diabetes diagnosis | Working level | Education level* |
| --- | --- | --- | --- | --- | --- | --- | --- |
| PCC1_P1 | Men | 50 | Spanish | Unknown | Yes | Employee | Low |
| PCC2_P2 | Women | 68 | Spanish | Metformin | No | Pensioner | Low |
| PCC2_P3 | Men | 60 | Spanish | Metformin | No | Unemployed | High |
| PCC3_P4 | Women | 60 | Spanish | Simvastatin | Yes | Employee | Medium |

CVD: Cardiovascular Disease; PCC: Primary Care Center.

*Education level: Primary education (low), secondary education (medium), and university education (high)

Supplementary Table 3. Discussion groups: Patients' characteristics

| **Patients** | **Sex** | **Age range** | **Nationality** | **Cardiovascular risk** | **Education level*** |
| --- | --- | --- | --- | --- | --- |
| **N: 15** | Women: 8  Men: 7 | <30: 1  31-60: 8  >61: 6 | Spanish: 14  Colombian: 1 | No: 8  Yes: 7 | Low: 2  Medium: 5  High: 8 |

*Education level: Primary education (low), secondary education (medium), and university education (high)

Supplementary Table 4. Clinical parameters values: Estimated Glomerular Filtration Rate Categorized.

| **Clinical parameter** | **Registries** (N) | **%** |
| --- | --- | --- |
| **Estimated Glomerular Filtration Rate MDRD** | **813** | 100% |
| Normal  (>60 mL/min/1.73m^2^) | 652 | 80.2% |
| Moderately decreased  (59-30 mL/min/1.73m^2^) | 142 | 17.47% |
| Severely diminished  (29-15 mL/min/1.73m^2^) | 14 | 1.72% |
| Advanced renal failure  (<15 mL/min/1.73m^2^) | 5 | 0.62% |
| **Estimated Glomerular Filtration Rate**  **CKD-EPI** | **816** | 100% |
| Normal  (>90 mL/min/1.73m^2^) | 304 | 37.25% |
| Slightly decreased  (60-89 mL/min/1.73m^2^) | 362 | 44.36% |
| Moderately decreased  (59-30 mL/min/1.73m^2^) | 127 | 15.56% |
| Severely diminished  (29-15 mL/min/1.73m^2^) | 15 | 1.84% |
| Advanced renal failure  (<15 mL/min/1.73m^2^) | 8 | 0.98% |
